# Supplementary material for: Whole genome resequencing of a laboratory-adapted Drosophila melanogaster population sample
Source: F1000Res. 2016 Dec 22;5:2644. Originally published 2016 Nov 7. [Version 3] doi: 10.12688/f1000research.9912.3 (PMC5115224; doi:10.12688/f1000research.9912.3)
Supplement: Supplementary file 1 [file f1000research-5-11365-s0000.tgz › 40694814-2c57-410c-ab49-60e87aee1111.pdf]

Supplementary information for: "Whole genome resequencing of a laboratory-adapted *Drosophila melanogaster* population"

William P. Gilks*[1], Tanya M. Pennell[1], Ilona Flis[1], Matthew T. Webster [2], Edward H. Morrow[1]

1. Evolution, Behaviour and Environment Group, School of Life Sciences, John Maynard Smith Building, University of Sussex, Falmer, BN1 9QG, United Kingdom, http://www.sussex.ac.uk/lifesci/morrowlab/ *w.gilks@sussex.ac.uk, wpgilks@gmail.com
2. Science for Life Laboratory, Department of Medical Biochemistry and Microbiology, PO Box 582, Uppsala Universitet, SE-751 23 Uppsala, Sweden

1 URLs for external data and Software

dm6 Reference assembly (GCA_000001215.4) ftp://hgdownload.cse.ucsc.edu/goldenPath/dm6/

FastQC 0.10.0 http://www.bioinformatics.babraham.ac.uk/

EA-Utils (cleaning of sequence reads) 1.1.2 https://code.google.com/p/ea-utils/

Burrows-Wheeler Aligner (BWA) 0.7.7-r441 http://bio-bwa.sourceforge.net/

Stampy 1.0.24 http://www.well.ox.ac.uk/project-stampy

Genome Analysis Tool-Kit (GATK) 3.2.2, and later 3.4-0, as specified in the code and main manuscript text. https://www.broadinstitute.org/gatk/

PicardTools 1.77 http://picard.sourceforge.net

SamTools 1.0 http://samtools.sourceforge.net/

GenomeStrip 2.0 http://www.broadinstitute.org/software/genomestrip/

Script for generating genotype calls from GenomeStrip/2.0 CNV likelihood scores. More recent versions of Genomestrip include this script. ftp://ftp.broadinstitute.org/pub/svtoolkit/misc/cnvs/estimate_cnv_allele_frequencies.R

2 Supplementary Tables

Table S1. Regions excluded from Genomestrip/2.0 structural variant results

Chromosome	Start position	Stop position	Feature
2L	0	20,000	telomere
2L	9,450,000	9,600,000	*In vivo* amplification
2L	13,300,000	13,500,000	*In vivo* amplification
2L	21,000,000	23,513,712	centromere
2R	0	6,000,000	centromere
2R	25,256,600	25,286,936	telomere
3L	0	70,000	telomere
3L	2,250,000	2,320,000	*In vivo* amplification
3L	8,500,000	8,800,000	*In vivo* amplification
3L	22,500,000	28,110,227	centromere
3R	0	4,500,000	centromere
3R	32,000,000	32,079,331	telomere
X	3,650,000	3,800,000	*In vivo* amplification
X	8,400,000	8,520,000	*In vivo* amplification
X	21,000,000	23,542,271	centromere

Genomic positions for centromic and telomeric regions were determined following visualisation of *bam* sequence alignment files, where the sequencing coverage was fragmented, causing read pairs to be excessively separated without evidence of structural variation.

Table S2. Structural variants called as multiple events by Genomestrip

Type	Chromosome	Start position*	Stop position*	Length(bp)	Sample present in
Duplication	2L	4,894,940	5,861,033	966,093	H037
Deletion	2L	15,335,536	16,655,783	1,320,247	H023
Deletion	2R	16,188,011	16,306,112	118,101	H029
Duplication	2R	21,499,905	22,386,557	886,652	H165
Deletion	3R	8,096,329	8,363,019	266,690	H111
Duplication	3R	15,720,028	17,043,150	1,323,122	H148
Duplication	3R	23,162,039	23,585,335	423,296	H050
Duplication	X	19,995,505	20,112,715	117,210	H203

*Start and stop positions were determined from the limits of individual events identified by Genomestrip. Positions are relative to the *D.melanogaster* reference assembly dm6.

3 Supplementary Figures

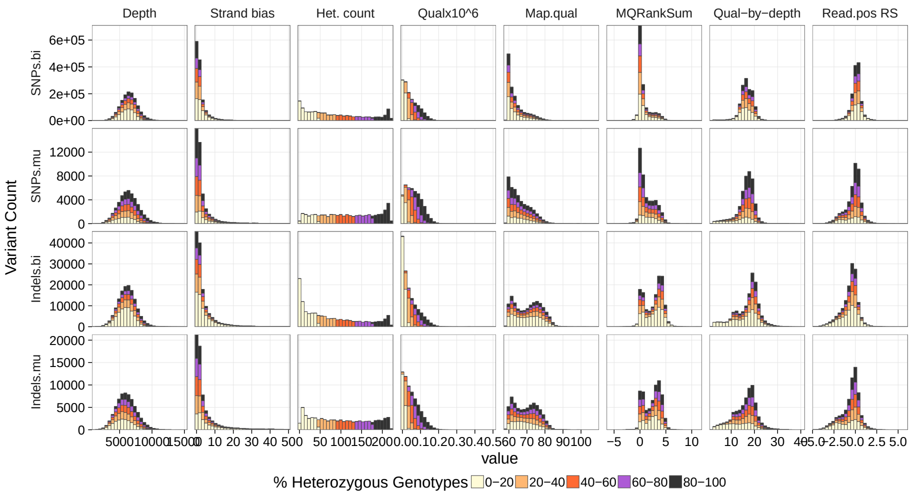

Figure S1. Haplotype Caller, distribution of quality metrics for SNPs and indels. Data generated by GATK VariantsToTable function and plotted in R/3.3.1. Plot bars are coloured by heterozygous genotype count, as a proxy for minor allele frequency in the hemiclone study sample. Code and data for this figure available at https://doi.org/10.5281/zenodo.159282.

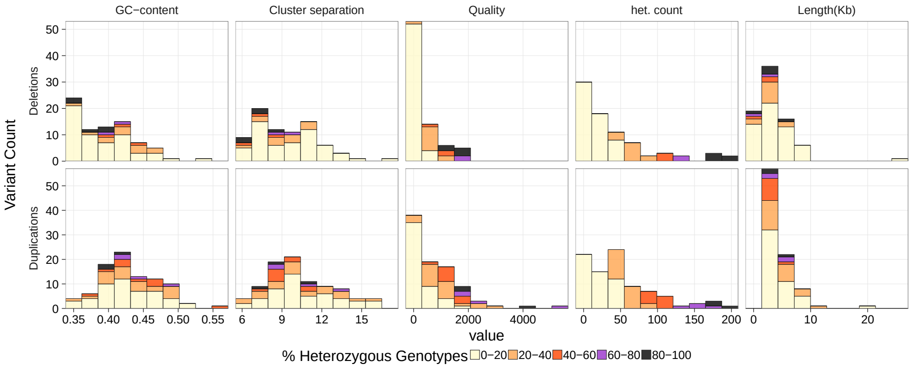

Figure S2. Genomestrip, distribution of quality metrics for structural variants. Data generated by GATK VariantsToTable function and plotted in R/3.3.1. Plot bars are coloured by heterozygous genotype count, as a proxy for minor allele frequency in the hemiclone study sample.Code and data for this figure available at https://doi.org/10.5281/zenodo.159282.

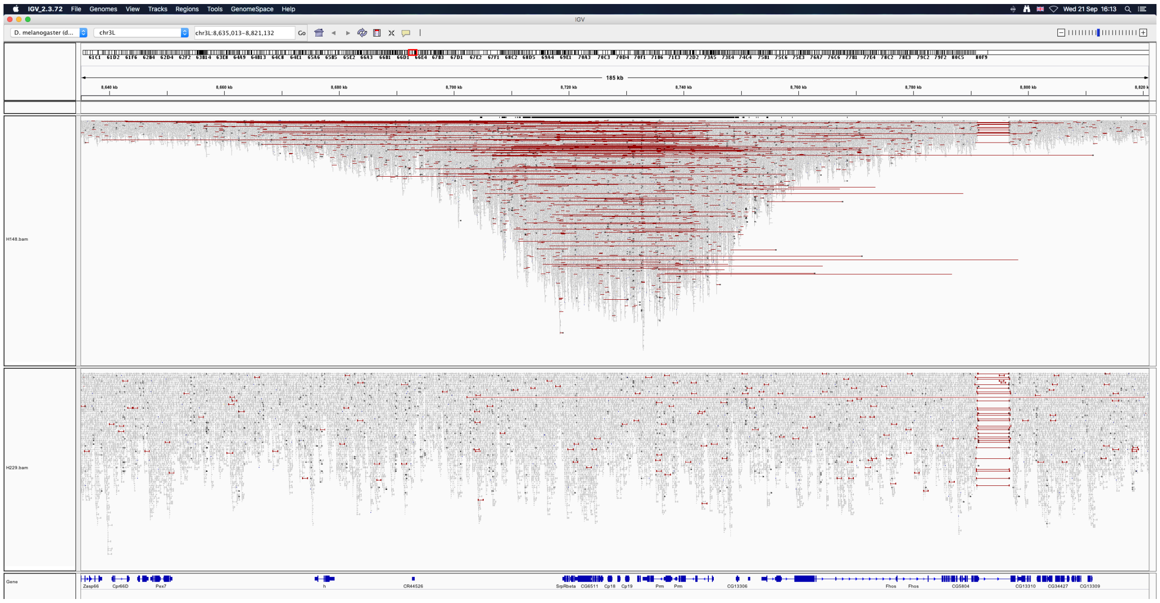

Figure S3. *In vivo* amplification. Example of likely *in vivo* amplification across chorion protein genes 18 and 19 on chromosome arm 3L. Image taken from visualisation of *bam* sequence alignment files using Integrated Genomics Viewer v2.3.72. Small grey blocks indicate sequence reads. Horizontal red lines indicate read pairs which are >1000bp apart. The upper sample (*H148*) exhibits the amplification, whereas the lower sample (*H001*) does not. Also shown below in dark blue, are the positions of genes in the region.
