# Supplementary material for: Whole genome resequencing of a laboratory-adapted Drosophila melanogaster population sample
Source: F1000Res. 2016 Dec 22;5:2644. Originally published 2016 Nov 7. [Version 3] doi: 10.12688/f1000research.9912.3 (PMC5115224; doi:10.12688/f1000research.9912.3)
Supplement: Peer review history

# Peer review: Referee response for version 1

**DOI:** 10.5256/f1000research.10683.r17452

**Published:** 2016-11-16

**Authors:** Geraldine A. Van der Auwera

**Affiliations:** 1 Data Science and Data Engineering group, Data Sciences Platform, Broad Institute of Harvard and MIT, Cambridge, MA, USA

Overall this is a very solid technical note. The methods seem sound, the descriptions are fairly straightforward, and caveats are properly acknowledged. The authors have provided detailed descriptions of what was done (including software tool versions) and made data and code available to reproduce not only the dataset but also all figures in the paper itself. 

 Regarding the experimental design, I think it's great that Gilks et al. chose to perform sequencing on individual flies rather than pooled samples. It takes some extra effort to deal with the small amounts of starting material involved, but the resulting dataset is that much more valuable. 

 It's also nice to see a study looking at CNVs and short variants together. As tools in this space improve and enable greater integration I look forward to seeing more analysis of how the different variant types relate to each other (e.g. looking at which short variants might be amplified or suppressed by CNV events). 

**Request for additional figures**

 I would recommend including diagrams of the hemiclonal experimental design and of the analysis workflows to maximize clarity. In particular, I think it could be made more obvious that the HaplotypeCaller workflow was run using the GVCF pathway for joint analysis. 

**Minor comments**

 I prefer "high-throughput sequencing" to "next generation sequencing" (this technology was "next-gen" ten years ago, now it's just the current standard). 

 On page 3, does "Fine mapping" refer to realignment around indels or equivalent processes? 

 On page 4, I would express "strand bias" as "FisherStrand estimation of strand bias" to avoid ambiguity with other estimators like Strand Odds Ratio, SOR).

 On page 4, does "null alternate alleles" refer to the GATK convention of emitting "*" to record sites with spanning deletions, as documented here: https://software.broadinstitute.org/gatk/guide/article?id=6926?

**Typos** (Page/paragraph)

 P2p3 - "detections" -> "detects"

 P2p6 - "off-spring" -> "offspring"

 P3p1 - "were were" -> "were"

 P4p1 - "mis-matches" -> "mismatches"

 P6p2 - "g.vcf" -> "GVCF" or "gVCF"

I have read this submission. I believe that I have an appropriate level of expertise to confirm that it is of an acceptable scientific standard.

# Response

**Published:** 2016-12-12

**Authors:** William Gilks

**Affiliations:** University of Sussex, UK

Dear Dr Van der Auwera,

 We thank you for reviewing our manuscript, and consider that your suggestions improve the clarity and quality of the manuscript, particularly for technical accuracy, and overall communication. Following these suggestions, we describe the changes that we have made:

1. As requested, we have included a figure describing the breeding of the hemiclonal lines and indicating what is happening to the chromosomes, (Figure1), including a reference to the figure in the main text.
2. As requested, we have included figure that summarises the analysis pipeline for the next-generation sequencing data and genotyping procedures (Figure 2).
3. In accordance with the suggestion, we have changed the term “next-generation sequencing” to "high-throughput sequencing". We have also added a reference to for the sequencing method (Introduction, first sentence, Bently et al 2008 Nature PMID:18987734).
4. We agree that our description of the sequence-read mapping procedures was generally vague and inaccurate (Page 3, Read Mapping methods, 2nd paragraph).
  Our original sentences were: “Fine mapping was performed with both Stampy v1.0.248 and the Genome Analysis Tool-Kit (GATK) v3.2.29 (following10). Removal of duplicate reads, indexing and sorting was performed with Picard- Tools v1.77 and SamTools v1.0.”
  We have changed this to: “Remaining reads were re-mapped using Stampy v1.0.24, which is slower but more precisely maps reads which are divergent from the reference genome assembly9. This method was used previously for the Drosophila Genome Nexus10. Removal of duplicate reads, indexing and sorting was performed with Picard-Tools v1.77. Re-mapping of sequence reads around insertion-deletion polymorphisms was performed using Genome Analysis Tool-Kit (GATK) v3.2.2, as a recommended standard practice11.”
  The updated text provides more information on the properties of secondary mapping using Stampy, and how it has been used previously for the Drosophila Genome Nexus. Furthermore, the new text, distinguishes the process of fine-mapping of reads around insertion-deletion polymorphisms using GATK.
5. You suggested for clarification, changing "strand bias" to "FisherStrand estimation of strand bias" in order to avoid ambiguity with other estimators (page 4, Small variant detection methods, 2nd paragraph, 3rd sentence).  We have added in brackets a definition for strand bias, in this case as ‘Phred-scaled p-value from Fisher’s Exact test’. We have also added this information to Table 1, on HaplotypeCaller variant metrics.
6. On page 4 (Small variant detection methods, last paragraph, preparation of data to NCBI dbSNP), you query whether our use of the term ‘null alternate allele’ as a reference to the GATK convention of using an asterisk symbol for an alternate allele which is located in a spanning deletion. We have removed the term ‘null alternate allele’, and merely stated that variants located within deletions were excluded.
  The original sentence was: “For data submission to dbSNP, we removed 44,644 indels that were multi-allelic or greater than 50bp in length, and a further 57,662 variants that had null alternate alleles (likely due to being situated within a deletion)."
  The new sentence is: “For data submission to NCBI dbSNP, we were obliged to exclude 44,644 indels that were multi-allelic or greater than 50bp in length, and a further 57,662 SNPs and indels situated within deletions.”
7. We have made the grammatical and spelling corrections as suggested.

We hope that these alterations meet your approval, and would be happy to make any further changes that may be required.

 Sincerely,

 William Gilks

 Tanya Pennell

 Ilona Flis

 Matthew Webster

 Edward Morrow

# Peer review: Referee response for version 1

**DOI:** 10.5256/f1000research.10683.r17453

**Published:** 2016-11-08

**Authors:** Stephen Richards

**Affiliations:** 1 Human Genome Sequencing Center, Department of Human and Molecular Genetics, Baylor College of Medicine, Houston, TX, USA

As a data note I think this is an excellent very detailed and comprehensive description of a dataset.

 I can find all the data in the public databases as described.

 I have only the most minor of quibbles:

1. The breeding of the hemiclonal lines always confuses me, and I think it would help the reader if there were a figure describing this with different colored chromosomes showing what is happening as you go through the crosses.
2. If I wanted a vcf file (or ideally gvcf) for the project is there one available for download.
3. Maybe stick the data in fly-var? http://www.iipl.fudan.edu.cn/FlyVar/

I have read this submission. I believe that I have an appropriate level of expertise to confirm that it is of an acceptable scientific standard.

# Response

**Published:** 2016-12-12

**Authors:** William Gilks

**Affiliations:** University of Sussex, UK

Dear Dr Richards,

 We thank you for reviewing our manuscript, and consider that your suggestions improve the quality of the manuscript, and the public availability of data. Following your suggestions, we have made the following changes:

1. As requested, we have included a figure describing the breeding design of the hemiclonal lines, which hopefully clarifies the transmission of the different chromosomes in each cross (Figure 1).
2. You suggested releasing a ‘gvcf’ file for public use, which contains a record of the genotype information at all sites in the D. melanogaster genome in the LHM population study sample. We had previously deleted this file after the smaller vcf file was generated. In response to the suggestion we have re-generated a gvcf, and deposited it in Zenodo (https://doi.org/10.5281/zenodo.198880), alongside code and run-logs, and made a note of this in the manuscript text under ‘Data Availability; Small variant data’. This gvcf differs from the original gvcf only in that: i) An updated version of GATK was used (3.4 compared to 3.2), and ii) That all scaffolds of the dm6 assembly were analysed, including those which have not been mapped to specific chromosomal positions.
3. You suggested that we deposit the genotype data in the ‘Fly-var’ database (http://www.iipl.fudan.edu.cn/FlyVar/). Following communication with the curators of Fly-var, we understand that the current method of data submission is by e-mail, which is unsuitable for our large dataset. We have given the URLs for our data to the Fly-var curators ready for upload when procedures exist.

We hope that these alterations meet your approval, and would be happy to make any further changes that may be required.

 Sincerely,

 William Gilks

 Tanya Pennell

 Ilona Flis

 Matthew Webster

 Ted Morrow
